# Radical Prostatectomy and Anatomical Controversies: The Urethral Sphincter and the Elusive Continence Mechanisms

**DOI:** 10.3390/cancers15133410

**Published:** 2023-06-29

**Authors:** Kostis Gyftopoulos

**Affiliations:** Department of Anatomy, School of Medicine, University of Patras, 26504 Patras, Greece; kogyftop@upatras.gr

Prostate cancer incidence is rising. This is especially true in younger age groups and may be partially attributed to the widespread use of PSA screening [1]. Although the exact etiology remains obscure, this rise may only lead to an increased number of radical prostatectomies [RP] performed worldwide. Since the initial descriptions of the techniques by HH Young almost 120 years ago and the revolutionary modifications by Walsh in the 1980s, many things have changed, resulting in substantial improvement in morbidity and mortality rates [2]. Yet, despite the innumerous modifications and the advent of laparoscopic and robotic surgery [RARP], it appears that the improvement of the technique has reached a plateau [2]. In the recent years, no further improvement in functional outcomes has been demonstrated and the expected rise in radical prostatectomies will eventually only translate to more men suffering from erectile dysfunction and—even worse—incontinence [2,3].

Although the post-prostatectomy incontinence (PPI) problem is multifactorial, the most common contributing factor to PPI is intrinsic sphincter deficiency (ISD), a damage to the striated male urethral sphincter, which most frequently occurs during the surgical manipulation of the prostatic apex [4]. This damage is most commonly myogenic and less often a denervation sphincteric injury. It is reasonable that the preservation of the sphincteric mechanism is of outmost importance for a “successful” radical prostatectomy. The deep knowledge of the respective pelvic anatomy certainly contributes to the application of surgical manipulations and orientation in the surgical field. However, the urethral sphincter appears to be an ongoing controversial issue when it comes to detailed anatomical descriptions. In most anatomy textbooks and atlases, the misconception of the striated external urethral sphincter (EUS) being a doughnut-shaped, circular component of the deep transverse perineal muscle (the muscle of the so-called *urogenital diaphragm*) is still present. This long-lasting misconception is based on a possible artifact in cadaveric dissection, described in the original work by Henle back in 1866 [5]. However, this misunderstanding still holds strong.

A possible explanation of this controversy (i.e., the nature of the male urethral sphincter) is based in the interpretation of the word *sphincter.* The origin of the word is ancient Greek (*σφιγκτήρ—sfiŋgtír*) and implies a “band, lace, anything that binds tight,” originating from the verb *σφίγγειν* (*sphingein*)—to squeeze or bind [6]. The application of the term in anatomy and medicine is attributed to Galen; however, the widespread notion that all sphincters are circular (*orbicularis)* is wrong; the interpretation of the word in Greek is much broader, suggesting a device that squeezes and compresses in more than one (circular) way (Figure 1).

Another possible rationalization of the controversies surrounding the external urethral sphincter may arise from the widespread use of cadaveric dissection in anatomical studies. Although valuable, cadaver use in exploring anatomy is prone to bias: most cadaveric dissections are performed on adults of older age, with the subsequent alterations in anatomy due to advanced age and subsequent tissue degeneration. The use of fresh cadavers is an option; however, alterations and interindividual variations in detailed sphincteric and prostate apical anatomy are still present, for instance due to concomitant benign prostatic hyperplasia [7,8]. Perhaps embryology is the “new” anatomy: embryological studies offer the possibility of characterizing the stages of developmental anatomy in detail, obviating the afore-mentioned obstacles.

The revolutionary work of Thomas Oelrich in 1980, which included dissection and microscopic evaluation in fetal and adolescent tissues, clearly defined that the urethral sphincter is not a muscular “ring” at the horizontal plane; instead, it is semicircular *funnel*, closely associated with the prostate and extending from the bladder to the perineal membrane [9]. Notably, Oelrich comments on the attempt to dissect a “typical cadaver of 50 years” as being an “effort fraught with difficulty and error due to the infiltration of connective tissue and the vasculature of the prostatic venous plexus” [9]. His concept of the male urethral sphincter has been verified by other embryological studies, which describe in detail the embryological origin of a common muscular primordium, which will eventually give rise to an inner, smooth muscle cylinder (internal urethral sphincter) and a striated, omega-shaped external urethral sphincter that is closed at a median raphe [10]. The prostate develops through budding from the dorsal surface of the urethra and grows into the overlying sphincteric muscle; this interaction sets the boundaries of both structures, i.e., the prostate and the sphincteric muscle (Figure 2).

However, this sphincteric complex cannot be viewed independently of the adjacent structures. As Oelrich emphasizes, it lies within the pelvic cavity and within the urogenital hiatus; other important structures are in close contact with it, share common innervation and have synergistic effects in the continence mechanism. The *levator ani* muscle (LAM) actually contributes to this mechanism through its pre-rectal fibers (often called *puboperinealis m*.), forming a sling that retracts the urethra towards the *symphysis pubis* area (Figure 3). The bilateral slings of the LAM component sandwich the striated EUS (rhabdosphincter) with the interposition of a connecting fascia, thus not only pulling the urethra (along with the EUS) towards the pubic bone but also compressing the urethral canal from the sides [11]. Interestingly, both the striated EUS and the pre-rectal fibers (*puboperinealis m.)* of the LAM are innervated by common pathways, including both pudendal and extrapudendal branches [12]. 

To complicate things even further, newer data support the presence of not only somatic but also autonomic (from the hypogastric and pelvic plexuses) pathways, which eventually arrange a coordinated role of the LAM and the EUS in the continence mechanism [12,13]. Last but not least, the smooth muscle *rectourethralis m*. may play a role in assisting the continence mechanism by resisting the traction from the rhabdosphincter and LAM on the urethra [11,13]. However, the disputable nature and action of this small muscle cannot be overemphasized: many authors question its direct connection to the urethra, as the *rectourethralis m*. is actually a misnomer, describing a bundle of smooth muscle fibers that derive from the outer longitudinal muscle of the midline rectal wall and are anchored to the perineal body distal to the prostate (hence the suggested anatomical term *rectoperinealis m*) [8]. Others even question the attachment to the median dorsal raphe of the EUS, suggesting that this raphe is not tendinous but contains elastic fibers and smooth muscles instead [11]. Nevertheless, while the role of this small muscle is debatable, it may however partially assist the stability of the urethral junction and may become part of the posterior reconstruction techniques offering dorsal support for the ureterovesical anastomosis, particularly at a level below the end of the Denonvilliers’ fascia (or *prostatoseminal vesicular fascia*), as the latter commonly ends at the rectourethralis muscle in males [11].

Apart from the Denonvilliers’ fascia, several layers and thickenings of the pelvic fascia invest and support the prostate gland and urethra complex. However, the fascial nomenclature is annoyingly complicated, as the same anatomical entity may be termed differently in textbooks, anatomical atlases and urological publications: the pelvic fascia has often been described as the lateral pelvic fascia, superior pelvic fascia, parietal pelvic fascia, *levator ani* fascia, outer layer of periprostatic fascia and parapelvic fascia, to name a few [14]. Perhaps the most helpful systematic view of the body fascias and, in particular, pelvic fascias, was described by Gallaudet in 1931—his definition of the *subcutaneous* and *subserous* systems facilitates the understanding of the complex distribution of the fascial system in the pelvic and perineal area [15]. Nevertheless, several portions of the (endo)pelvic fascia seem to play a crucial role in the support and stabilization of the urethra; this is especially evident for the puboprostatic (or, more appropriately, the pubovesical) ligaments but also for the connective tissue supporting the aforementioned thickenings of the pelvic fascia [16]. Moreover, the fascial system should be viewed more like a complex framework of connective tissue than simple sheets of membranes. The impairment of this scaffold may be the result of surgical manipulations but may also be due to the simple enlargement of the prostate gland, secondary to BPH. This deterioration affects the stability of the urethra and sphincter, weakens the contraction force of the relative muscles, and may also result in the hypermobility of the urethra, adding more factors into the post-prostatectomy incontinence issue [17]. It is now generally accepted that the techniques of both posterior and anterior musculofascial restoration during RP may result in better continence results, at least in the early postoperative phase [4,18,19].

The evolution of our knowledge on the detailed, applied anatomy of the sphincteric complex of the male urethra represents a paradigm shift of how new data from anatomical, clinical and embryological studies are incorporated into the technical improvements of a now common surgical procedure, such as radical prostatectomy. A variety of modifications of the surgical technique have been developed, based on the anatomical changes of the pelvic and perineal structures, aiming at an improved oncological and functional outcome [20]. It would be interesting to identify the time lag necessary for these elucidations of classical anatomical controversies to become incorporated into modern anatomy textbooks and atlases, which not only remain a cornerstone for undergraduate studying but also represent a common resource material for surgical trainees and surgeons worldwide [21].

This Special Issue is dedicated to other similarly controversial anatomical and surgical debate topics in radical prostatectomy. Can we predict the incidence of incontinence after RP? A useful nomogram developed by Pinkhasov et al. yields a model superior to any single clinical variable for predicting the risk of incontinence after RARP [22]. Does the size of the prostate gland affect the outcome of the procedure? It appears that larger prostate size is associated with increased blood loss, a higher rate of complications but with better oncological outcome, as described in the systematic review by Fahmy et al. [23]. Is there any specific role to the preservation of Denonvilliers’ fascia or is it just a simple sheet of tissue with no clinical significance? [24]. Do different nerve-sparing techniques have an actual impact on the clinical and functional outcomes of RP? [25]. Additionally, what do “positive margins” in the prostatectomy specimen mean for the clinician? Is marginal invasion always predictive of patients’ clinical outcome? [26]. These interesting questions are presented in this Special Issue in an attempt to shed light to long-standing debates in RP.

## Figures and Tables

**Figure 1:**
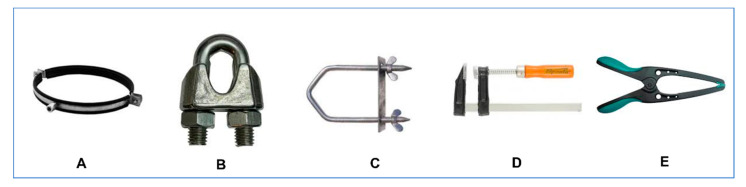
Several types of mechanical sphincters (*σφιγκτήρες)* with circular (**A**), semi-circular, omega shaped (**B**,**C**) and side sphincteric action (**D**,**E**).

**Figure 2:**
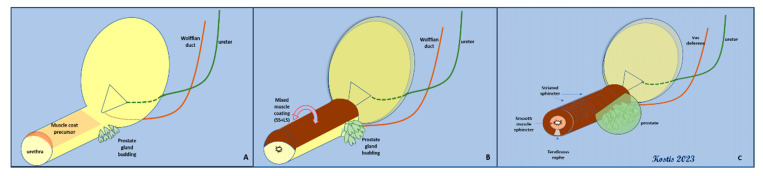
Embryological development of both elements of the urethral sphincter (LS, smooth muscle–lissosphincter, and SS, striated muscle–external sphincter) from a common muscular primordial plate. (**A**,**B**): the initial covering of the urethra with the concomitant budding of prostate glands. (**C**): the final arrangement of the bilayered sphincter around the urethra—the development of the prostate gland has reached its limit (SS) at the dorsal surface.

**Figure 3:**
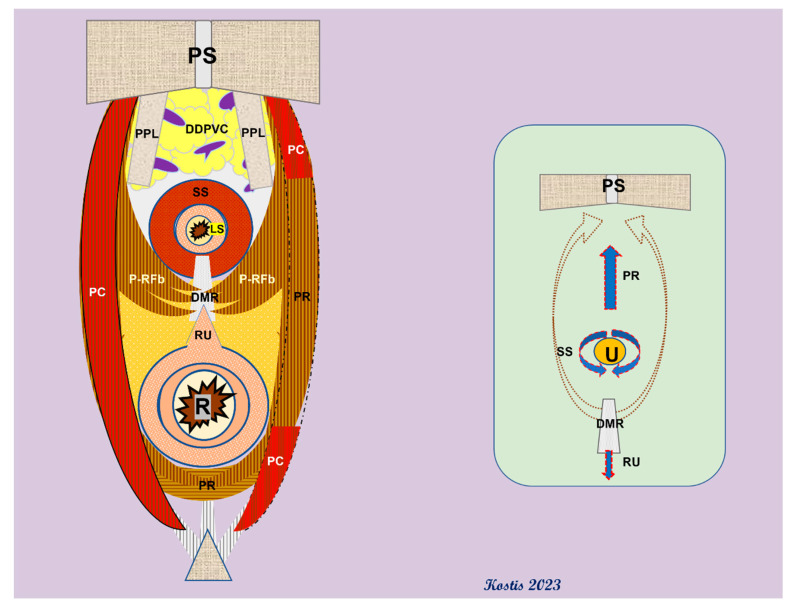
Arrangement of the pre-rectal fibers of the *puborectalis m* (also: *puboperinealis m*.) around the urethral hiatus. *Inset: net forces applied to the urethra lumen by muscle contractions (red arrows)*. PS: pubic symphysis; PPL: puboprostatic ligaments; PC: *pubococcygeus m*.; DDPVC: deep dorsal penile vein complex; PC: P-RFb: pre-rectal fibers of the PR; DMR: dorsal median raphe; *PR: puborectalis m*; RU: *rectourethralis m*.; SS: striated sphincter; LS: lissosphincter; U: urethra; R: rectum.
